# Does hypericin boost the efficacy of high-power laser? A preliminary experimental study on rats

**DOI:** 10.1590/1516-3180-2014-1326625

**Published:** 2014-09-02

**Authors:** Murat Zor, Serdar Goktas, Ibrahim Yildirim, Unal Orhan Zorba, Seref Basal, Bilal Firat Alp, Engin Kaya, Osman Erogul

**Affiliations:** I MD. Urology Specialist, Department of Urology, Sarikamis Military Hospital, Kars, Turkey; II MD. Professor and Head of Department of Urology, Selcuklu Medical Faculty, Selcuk University, Konya, Turkey; III MD. Professor, Department of Urology, Gülhane Military Medical Academy, Ankara, Turkey; IV MD. Associate Professor, Department of Urology, Rize University Medical Faculty, Rize, Turkey; V MD. Associate Professor, Department of Urology, Gülhane Military Medical Academy, Ankara, Turkey; VI MD. Urology Specialist, Department of Urology, Gülhane Military Medical Academy, Ankara, Turkey; VII PhD. Head of Department of Biomedical Science, Gülhane Military Medical Academy, Ankara, Turkey

**Keywords:** Hypericum, Lasers, solid-state, Prostatic hyperplasia, Photochemotherapy, Endoscopy, Hypericum, Lasers de estado sólido, Hiperplasia prostática, Fotoquimioterapia, Endoscopia

## Abstract

**CONTEXT AND OBJECTIVE:**

Lasers are widely used in treating symptomatic benign prostatic hyperplasia. In current practice, potassium titanyl phosphate (KTP) lasers are the most common type of laser systems used. The aim here was to evaluate the rapid effect of high-power laser systems after application of hypericin.

**DESIGN AND SETTING:**

Experimental animal study conducted in the Department of Urology, Gülhane Military Medical Academy, Ankara, Turkey, in 2012.

**METHODS:**

Sixteen rats were randomized into four groups: 120 W KTP laser + hypericin; 120 W KTP laser alone; 80 W KTP laser + hypericin; and 80 W KTP laser alone. Hypericin was given intraperitoneally two hours prior to laser applications. The laser incisions were made through the quadriceps muscle of the rats. The depth and the width of the laser incisions were evaluated histologically and recorded.

**RESULTS:**

To standardize the effects of the laser, we used the ratio of depth to width. These new values showed us the depth of the laser application per unit width. The new values acquired were evaluated statistically. Mean depth/width values were 231.6, 173.6, 214.1 and 178.9 in groups 1, 2, 3 and 4, respectively. The most notable result was that higher degrees of tissue penetration were achieved in the groups with hypericin (P < 0.05).

**CONCLUSIONS:**

The encouraging results from our preliminary study demonstrated that hypericin may improve the effects of KTP laser applications.

## Abstract (pt)

**CONTEXTO E OBJETIVO:**

Lasers são amplamente utilizados no tratamento de hiperplasia benigna de próstata sintomática. Na prática atual, lasers de fosfato de titanilo de potássio (KTP) são os tipos mais comuns usados dos sistemas. O objetivo foi avaliar o efeito rápido do sistema laser de alta potência após a aplicação de hipericina.

**TIPO DE ESTUDO E LOCAL:**

Estudo experimental animal, realizado no Departamento de Urologia, Academia de Medicina Militar de Gülhane, Ancara, Turquia, em 2012.

**MÉTODOS:**

16 ratos foram divididos aleatoriamente em 4 grupos: 120W KTP laser + hipericina; 120W KTP laser somente; 80W KTP laser + hipericina; 80W KTP laser somente. Hipericina foi dada intraperitonealmente duas horas antes da aplicação do laser. As incisões a laser foram feitas através do músculo quadríceps dos ratos. A profundidade e a largura das incisões a laser foram avaliadas histologicamente e registradas.

**RESULTADOS:**

Para padronizar o efeito do laser foi utilizada a razão entre profundidade e largura. Estes novos valores nos mostraram a profundidade da aplicação do laser de largura por unidade. Os novos valores adquiridos foram avaliados estatisticamente. Os valores da média de profundidade/largura foram 231,6, 173,6, 214,1 e 178,9 nos grupos 1, 2, 3 e 4, respectivamente. O resultado mais notável foi atingir altos graus de penetração tecidual nos grupos com hipericina (P < 0,05).

**CONCLUSÕES:**

Os resultados promissores do nosso estudo preliminar mostraram que hipericina pode melhorar os efeitos das aplicações do laser KTP.

## INTRODUCTION

Lasers are widely used in urological surgery, especially for man agement of symptomatic benign prostate hyperplasia (BPH). The major advantages of light-induced tissue removal are that it vaporizes and coagulates at the same time. These characteris tics of lasers simplify the treatment of symptomatic BPH both for surgeons and for patients. 

Potassium titanyl phosphate (KTP) lasers are based on systems that produce neodymium-YAG laser light; this light is then passed through a KTP crystal. This doubles the frequency and halves the wavelength of the laser light emitted to 532 nm, which is in the green part of the visible spectrum. Historically, application of KTP laser energy in urology began in the late 1980s when the early KTP laser systems with power outputs of initially 20 W and later 38 W became available.^1^ However, it took a long time for KTP lasers to be developed to the point of reaching sufficient power to vaporize the prostate rapidly. The 120 W KTP laser introduced by Laserscope-AMS in 2006 as the GreenLight HPS had the benefit of greater vaporization capability and decreased duration of surgery.^2^ However, high power brings some risks such as the potential for inadvertently penetrating too deeply with the laser and causing perforation of the bladder, injury of the orifice, especially with large median lobes, or perforation of the prostatic capsule with opening of large venous sinusoids at the end of the procedure. To achieve accurate laser power for rapid vaporization while avoiding harm to the adjacent tissues is a hard balance to maintain. 

Hypericin is a naturally occurring polycyclic aromatic naphthodianthrone that is isolated from plants of the genus *Hypericum*. This natural substance has been used in traditional Chinese medicine for thousands of years. In recent time, teas made from plants containing hypericin (St. John's wort) have been reported to have antineoplastic, antiviral and antidepressant properties.^3^^,^ 4

Photodynamic therapy (PDT) involves activation of a photosensitizer by means of light of an appropriate wavelength to produce focal damage to tissue. The treatment typically involves systemic administration of a photosensitizer and its subsequent activation by light of an appropriate wavelength to create a photochemical reaction that causes photodamage. PDT has become an accepted treatment for certain types of malignant tumors and is showing great promise for treating and managing a variety of nonmalignant diseases.^5^

Hypericin contains extended pi-orbital electrons that become excited by ultraviolet and green light, thus leading to production of toxic singlet oxygen and radical species.^6^^-^ 8

VanderWerf et al. were the first to report that KTP lasers and hypericin presented strong phototoxicity towards human tumor cell lines.^9^ Both apoptosis and necrosis have been proposed as mechanisms for cell death resulting from hypericin PDT. The photocytotoxic activity of hypericin has been tested in several experimental studies *in vivo* and *in vitro*.^10^^-^ 14

Hypericin is reported to be safe because no toxic effects on mice kept in the dark have been observed, nor have there been any genotoxic effects from a variety of *in vitro* and *in vivo* assays.^15^^,^ 16 Moreover, hypericin is present as a constituent in *Hypericum* extract, which is used clinically as an antidepressant and apparently causes no side effects.^17^ Taken together, these attributes make hypericin a potential tool in PDT.

Recent clinical testing has revealed that hypericin was not tumoricidal in a patient with mesothelioma who was exposed to 632 nm red laser light, but clear evidence of phototoxicity was seen in two subjects after intradermal drug injection when activated by green laser emissions at 514 nm rather than by 632 or 670 nm red light.^18^^,^ 19

Obstructive tissue is instantly removed by vaporization, but laser energy that is not absorbed by the superficial tissue will penetrate deeper, thus causing a rim of 1-2 mm of coagulated prostatic tissue and residual stromal tissue inside the prostatic surgical capsule. By using hypericin, KTP laser energy could be absorbed more efficiently.

To our knowledge, studies on the photodynamic therapeutic properties of hypericin have only focused on the late immunological response of tumor tissue to low-power laser and no study on high-power laser has been published on the subject. None of the observers evaluated the rapid effect of high-power laser systems after application of hypericin as a photosensitizer.

## OBJECTIVE

To evaluate the rapid effect of high-power laser systems after application of hypericin and whether this can be adopted for laser vaporization of the prostate. 

## METHODS

This study was planned as a preliminary study and was con ducted after obtaining approval from the local committee of our Medical Academy. All animal experiments were done in accordance with the Principles of Laboratory Animal Care (prepared by the National Institutes of Health; NIH Publication No. 85-23 rev. 1995). 

Sixteen male Wistar rats weighing 300-350 g were randomized into four groups of four animals each: 1) 120 W KTP laser with hypericin application; 2) 120 W KTP laser alone; 3) 80 W KTP laser with hypericin application; and 4) 80 W KTP laser alone. Hypericin at a dose of 1 µg/g (Deutsche Homöopathie-Union, DHU, Karlsruhe, Germany) was given intraperitoneally two hours prior to laser applications. 

**Figure 1:**
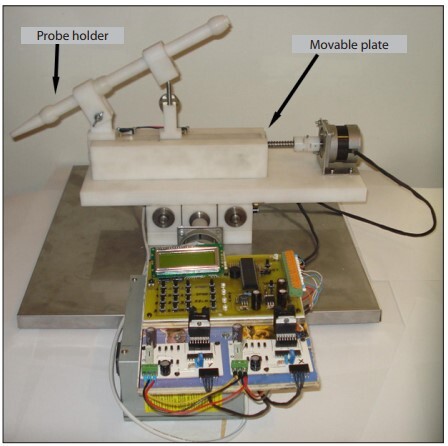
Laser movement system.

The animals were anesthetized by means of intraperitoneal injection of a mixture of ketamine hydrochloride (50 ml/kg) and xylazine hydrochloride (5 ml/kg). The animals were then placed in a ventral position and fixed. The skin above the biceps femoris muscle was dissected until the sole biceps femoris muscle had been exposed. Laser incisions were made through the biceps femoris muscle of the rats. To achieve standardization of the laser incisions, we used a "laser movement system" (Figure 1). With this device, we gained the ability to move the laser probe at a constant velocity over equal distances in the X and Y planes. Through this, the laser probes produced standardized incisions. During laser operation, the bare fiberoptic was adjusted to move at 4 mm/sec, parallel to and 3 mm above the denuded biceps femoris muscle. The incision length was 1.5 cm. After laser application, the biceps femoris muscle was dissected and was fixed in 10% neutral buffered formalin for 24 hours and then embedded in paraffin. The depth and width of the laser incisions were evaluated histologically and recorded. To assess and standardize the effects of the laser, we used the ratio of depth to width. These new values showed us the depth of the laser application per unit width. The new values acquired were evaluated statistically.

### Statistical analysis

The statistical data were analyzed using the Statistical Package for the Social Sciences (SPSS, Chicago, IL, USA), version 15.0 for Windows. All values were expressed as mean ± standard devia tion (SD). The differences between means were analyzed by using the Mann-Whitney U test. P values less than 0.05 were consid ered to indicate statistical significance. 

## RESULTS

The most notable result from the study was that higher degrees of tissue penetration were achieved in the groups with hypericin. This difference was statistically significant (P < 0.05). The mean depth/width values and the statistical analysis are summarized in Table 1. 

**Table 1:** Comparison of mean depth/width values between the groups with the Mann-Whitney U test

Groups	Depth/width	P-value
Group 1 (hypericin + 120 W KTP laser)	231.6 ± 38.9	P < 0.05
Group 2 (120 W KTP laser alone)	173.6 ± 52.5	
Group 3 (hypericin + 80 W KTP laser)	214.1 ± 47	P < 0.05
Group 4 (80 W KTP laser alone)	178.9 ±7.6	

## DISCUSSION

One of the cardinal rules for photoselective vaporization of the prostate (PVP) is that the surgeon needs to see a good cav ity, created with an open channel from the verumontanum to the bladder. PVP relies on instant vaporization rather than on delayed tissue sloughing. Therefore, "what you see" at the end of the procedure is exactly "what you will get".^2^ Studies on the mechanism of tissue responses to PDT in vivo have revealed that the tissue destruction may be either an immediate effect due to direct cell killing or a late effect due to vascular dam age and immunoreactions.^5^^,^ 20 ^,^ 21 Most previous studies focused on low-energy lasers and observed cell death after a couple of hours; none of the studies used high-power laser systems with application of hypericin.^10^^,^ 22 ^-^ 25 Use of low-power KTP will result in early edema and delayed sloughing of necrotic tissue conse quent to vascular damage and immunoreactions, and more so than the direct tissue ablation mentioned above, thereby result ing in prolonged obstruction and severe postoperative dysuria. Because of these limitations, we used high-power KTP in our study. To our knowledge, this is the first study on the use of hypericin with high-power KTP laser. 

The photoselective properties of the green 532 nm laser wavelength arise because this visible wavelength is strongly absorbed within the very superficial layer of tissue, given that the chromophores of the blood vessels and hemoglobin contained in this layer serve as primary and selective absorbers. Oxyhemoglobin has maximum excitation capacity at 414 nm wavelength.^20^^,^ 26 Hypericin is also an appropriate absorber for KTP laser, since its maximum excitation occurs at 545 nm wavelength.^2^^,^ 19

The time interval between administration of hypericin and application of the laser plays an important role regarding drug distribution between vessels and tissue. Half an hour after administration, intense hypericin fluorescence is mainly observed within blood vessels, with a small amount of fluorescence in the perivascular region.^25^^,^ 27 ^,^ 28 This maximizes the absorption of the laser energy in the vasculature. The peak concentration of hypericin in tissue is reached after four hours. Six hours after administration, only faint fluorescence is observed in the vasculature.^24^

Use of laser energy within 30 minutes after application of hypericin, i.e. at the time when it mostly is still in the vessels, does not provide any extra gain in relation to the classical PVP because KTP laser is instantly and nearly completely absorbed in blood. Waiting more than four hours after hypericin administration would result in dissemination of hypericin into the prostate. The depth of KTP laser penetration into the prostate is 1 mm.^26^ This is a limitation on investigating the effectiveness of hypericin, given that KTP laser energy is absorbed mostly by the hemoglobin in well-vascularized prostate tissue. In order to reach equilibrium between tissue and vasculature, we applied the laser two hours after administration of hypericin. 

In the literature, there are several studies showing statistically significant differences in necrosis between rats with and without hypericin. However, none of these studies used powers higher than 1 W.^9^^-^ 11 ^,^ 22 ^-^ 24 In our study, we used laser energies of 80 and 120 W, and to standardize the laser effectiveness we used the ratio of depth to width. We found that with hypericin administration, the tissue penetration of the green light laser was greater and reached a statistically significant difference.

The results obtained from this preliminary study are promising for future studies. Development of novel hypericin-containing drugs may decrease the frequencies of complications and morbidity from laser vaporization of the prostate, through reducing the duration of the operation, and may lead to greater efficiency of the operation. These results are encouraging with regard to shortening the duration of the operation and increasing the effectiveness of laser prostate operations in the future. Nonetheless, further studies are warranted in order to determine the value of this approach, with a view to introducing it within clinical scenarios.

## CONCLUSIONS

The results of our study demonstrated higher tissue penetration levels through hypericin applications. To our knowledge, this is the first study evaluating the rapid effect of high-power laser sys tems after application of hypericin as a photosensitizer. 
